# Tumor cell plasticity and intrinsic immunogenicity: Implications for immunotherapy resistance in small‐cell lung cancer

**DOI:** 10.1111/1759-7714.14117

**Published:** 2021-08-31

**Authors:** Ji Ruan

**Affiliations:** ^1^ State Key Laboratory of Oncology in South China Collaborative Innovation Center for Cancer Medicine, Sun Yat‐sen University Cancer Center Guangzhou China

Lung cancer is the most common cancer and the leading cause of cancer death in the world, accounting for 2.21 million new cases and 1.80 million deaths in 2020.^1^ China contains about 20% of the world population, but accounts for nearly 40% of all newly diagnosed lung cancer cases and deaths.^2^ The burden of lung cancer in China is very high and unfortunately it will increase further.^3^ Lung cancers can be divided into non‐small‐cell lung cancer (NSCLC) and small‐cell lung cancer (SCLC). NSCLC has been determined to be the predominant subtype of lung cancer, but the most lethal lung malignancy is SCLC. Most SCLC patients develop drug resistance soon after receiving traditional chemotherapy, resulting in a very low 5‐year overall survival rate.^4^ Although the tumor mutation burden of SCLC is high, the addition of immune checkpoint blockade to chemotherapy rarely results in a durable treatment response.^5^

SCLC is classified into two subtypes: neuroendocrine and non‐neuroendocrine. The neuroendocrine subtype shows a classical neuroendocrine morphology and high expression of the transcriptional regulators, such as ASCL1 and NEUROD1. The non‐neuroendocrine subtype shows a mesenchymal morphology and high expression of transcription factors such as c‐Myc, POU2F3, and YAP1, but downregulates ASCL1 and neuroendocrine markers (synaptophysin and chromogranin A).^6^ Previous studies based on SCLC genetically engineered mouse models have shown that neuroendocrine and non‐neuroendocrine tumor cells coexist in tumor mass, and these two subtypes of tumor cells can be transformed into each other.^7^

One of the key steps of immunosurveillance is the presentation of peptides on the major histocompatibility complex class I molecules (MHC I) to CD8^+^ T cells.^8^ The major mechanism of immune escape adopted by tumor cells is to reduce antigen presentation by suppressing the expression of MHC I. In tumor cells, MHC I‐mediated alterations in immunosurveillance can occur through either genetic or epigenetic means, and can affect any component of the MHC I antigen processing and antigen presentation mechanism (APM).^9^ SCLC has poor immunogenicity and exhibits low inherent levels of MHC I, which is considered to be the result of epigenetic programming.^10^ However, few studies have previously analyzed the intersection of MHC I‐mediated antigen presentation and SCLC heterogeneity.

In recent years, multiplex immunofluorescence (mIF) to simultaneously detect multiple proteins in the same tissue section emerged as very powerful tools to study the cellular and immunologic heterogeneity of tumor.^11^ In a study recently published in *Cancer Discovery*, titled “Intrinsic immunogenicity of small cell lung carcinoma revealed by its cellular plasticity”, Mahadevan et al.^12^ profiled a large set of SCLC tissues using both mIF and standard chromogenic immunohistochemistry (IHC) to explore the tumor heterogeneity and immune contexture of SCLC. A total of 102 SCLC cases obtained from multiple commercial tissue suppliers in the United States were examined. The results showed that MHC I^high^ SCLC tumors exhibited a non‐neuroendocrine morphology and had a durable response of immune checkpoint blockade. To further explore the cell state plasticity sufficient to allow conversion between neuroendocrine and non‐neuroendocrine tumor cells and the potential epigenetic mechanism of cellular plasticity in SCLC, the author analyzed the SCLC NCI‐H69 (H69) cells and their non‐neuroendocrine subpopulation (H69M) by H3K27 chromatin immunoprecipitation sequencing (ChIP‐seq) and RNA sequencing (RNA‐seq). They found that depression of *TAP1* epigenetic silencing can lead to the transition of SCLC cells from the neuroendocrine state to the non‐neuroendocrine state and the recovery of MHC I‐mediated antigen presentation. Additionally, the results of ChIP‐seq suggest that EZH2 can regulate the epigenetic plasticity of SCLC. Indeed, the following in vitro experiments demonstrated that inhibition of EZH2 promoted the neuroendocrine to non‐neuroendocrine transition, which leads to MHC I recovery. Consistent with these in vitro findings, the authors found that non‐neuroendocrine SCLC tumors were rejected in a syngeneic mouse model, which was manifested by the clonal expansion of CD8^+^ T cells with immunodominant effectors. Moreover, EZH2 inhibition combined with STING agonism increased the ability of T cells to recognize tumor cells in mice, resulting in the enhancement of SCLC rejection and improvement of overall survival.

This study uncovered a non‐neuroendocrine, MHC I^high^ SCLC subpopulation with intrinsic immunogenicity for the first time and found that this subpopulation had durable response to immune checkpoint blockade. This finding is supported by a recent study on the identification of inflamed SCLC subtypes,^13^ indicating that programmed death‐ligand 1 (PD‐L1) expression of immune cells is a key immunosuppressive mediator in this context. Furthermore, the overall survival rate of SCLC is still very limited, even if the addition of immune checkpoint blockade improves the response rate of first‐line treatment. Therefore, it is very important to develop new markers for accurately distinguishing immune checkpoint blockade responders from nonresponders. Unfortunately, unlike NSCLC, the expression level of PD‐L1 in SCLC is generally low, thus PD‐L1 expression cannot be used to predict immune checkpoint blockade response in SCLC.^14^ Tumor mutation burden (TMB) can predict the response of immune checkpoint blockade to a certain extent, but SCLC is closely associated with smoking, the TMB of SCLC is generally high, so the prediction accuracy of TMB is low.^15^ In contrast, MHC I is one of the targets of CD8^+^ T cells reactivated by immune checkpoint blockade. Therefore, it is very possible that the expression of MHC I can accurately distinguish those SCLC patients who can develop a durable response to immune checkpoint blockade. Of course, this hypothesis needs larger cohorts of SCLC patients to be verified in the future.

Additionally, the epigenetic and protein expression profiling of the HLA^+^ cell line and SCLC tissues in this study provided a description of the MHC I^high^ phenotype of SCLC. MHC I^high^ SCLC neither expresses neuroendocrine markers, such as NEUROD1 and ASCL1, nor expresses markers of the non‐neuroendocrine state, such as c‐Myc and YAP1. It highly expresses the epithelial‐mesenchymal transition (EMT) marker AXL. The recently identified inflamed SCLC subtype has similar transcriptional characteristics to the MHC I^high^ subtype: the upregulation of AXL and immune‐associated genes.^13^ This evidence reveals that the upregulation of AXL is an important feature of the non‐neuroendocrine state of SCLC.

Furthermore, this study revealed that epigenetic characteristics are major regulators of immunologic plasticity in SCLC as well as TAP1 and STING governs MHC I‐mediated antigen presentation directly. Last but not least, this study explored the rationale for combining EZH2 inhibitors with STING agonists in syngeneic animal models. The regulation of MHC I expression by EZH2 has been reported in SCLC previously.^10^ In addition to reconfirming this finding, this study also demonstrated that the transition of SCLC from the neuroendocrine to the non‐neuroendocrine cell state leads to increased expression of MHC I‐restricted antigens, and ultimately leads to antigen‐specific T‐cell‐mediated antitumor response in a syngeneic system. It is worth noting that the immunodominant clones of T cell identified in this study recognize the putative MHC I‐restricted antigens only expressed on non‐neuroendocrine SCLC cells, which indicates that epigenetic modulation is the molecular mechanism of immunopeptidome repertoire plasticity. Indeed, the authors of this study have previously discovered a unique endogenous retroviral suppression effect in this context,^7^ which indicates that in addition to more canonical tumor‐associated antigens or mutated neoantigens, specific retro‐element‐derived peptides may be the source of such immunogenicity.

Overall, the most important finding of this study is that EZH2 inhibition can transform neuroendocrine SCLC into an antigenic cell state that is responsive to STING stimulation in vivo. This finding suggests that future clinical studies of SCLC should focus on the combination of EZH2 inhibitors and STING agonists to overcome the inherent immunotherapy resistance of the neuroendocrine state of SCLC. In addition, the identification of non‐neuroendocrine subpopulations in human SCLC through cell state or immunological analysis may also help to stratify patients, which has guiding significance for the formulation of SCLC immunotherapeutic strategies.

## CONFLICT OF INTEREST

The author declares no competing interests.
